# A novel azithromycin resistance mutation in *Mycoplasma genitalium* induced *in vitro*

**DOI:** 10.1093/jac/dkaf174

**Published:** 2025-06-04

**Authors:** Teck-Phui Chua, Jennifer Danielewski, Catriona S Bradshaw, Dorothy A Machalek, Suzanne M Garland, Jose L Huaman, Jørgen S Jensen, Gerald L Murray

**Affiliations:** Department of Obstetrics, Gynaecology, and Newborn Health, University of Melbourne, Parkville, Victoria, Australia; Centre for Women’s Infectious Diseases, The Royal Women’s Hospital, Parkville, Victoria, Australia; Molecular Microbiology Research Group, Murdoch Children’s Research Institute, Parkville, Victoria, Australia; Centre for Women’s Infectious Diseases, The Royal Women’s Hospital, Parkville, Victoria, Australia; Molecular Microbiology Research Group, Murdoch Children’s Research Institute, Parkville, Victoria, Australia; Melbourne Sexual Health Centre, Alfred Health, Carlton, Victoria, Australia; School of Translational Medicine, Monash University, Melbourne, Victoria, Australia; Centre for Epidemiology and Biostatistics, Melbourne School of Population and Global Health, University of Melbourne, Parkville, Victoria, Australia; The Kirby Institute, University of New South Wales, Sydney, New South Wales, Australia; Department of Obstetrics, Gynaecology, and Newborn Health, University of Melbourne, Parkville, Victoria, Australia; Centre for Women’s Infectious Diseases, The Royal Women’s Hospital, Parkville, Victoria, Australia; Molecular Microbiology Research Group, Murdoch Children’s Research Institute, Parkville, Victoria, Australia; Department of Obstetrics, Gynaecology, and Newborn Health, University of Melbourne, Parkville, Victoria, Australia; Centre for Women’s Infectious Diseases, The Royal Women’s Hospital, Parkville, Victoria, Australia; Molecular Microbiology Research Group, Murdoch Children’s Research Institute, Parkville, Victoria, Australia; Research Unit for Reproductive Microbiology, Statens Serum Institut, Copenhagen, Denmark; Department of Obstetrics, Gynaecology, and Newborn Health, University of Melbourne, Parkville, Victoria, Australia; Centre for Women’s Infectious Diseases, The Royal Women’s Hospital, Parkville, Victoria, Australia; Molecular Microbiology Research Group, Murdoch Children’s Research Institute, Parkville, Victoria, Australia

## Abstract

**Background:**

*Mycoplasma genitalium* is a sexually transmitted bacterium of increasing concern due to issues around antimicrobial resistance. Resistance is typically mediated by SNPs; however, the difficulty of isolation and culture of *M. genitalium* limits the ability to analyse the impact of individual mutations.

**Objectives:**

The aim of this study was to generate and characterize antibiotic-resistant *M. genitalium* mutants *in vitro* to understand the development of macrolide resistance in this bacterium.

**Methods:**

Sequential MIC assays for azithromycin were performed using the laboratory strain of *M. genitalium* (G37) grown in Hayflick medium. Bacteria were enumerated by droplet digital PCR (ddPCR) targeting *mgpB*, and a new ddPCR assay was established to detect specific mutations in the 23S rRNA gene. MICs of selected macrolide antibiotics were determined in Hayflick medium. Whole genome sequencing (WGS) was performed on the Oxford Nanopore MinION.

**Results:**

After eight passages in azithromycin, a novel 23S rRNA gene mutation, G2057A (*Escherichia coli* numbering), was detected. The mutant did not display a detectable growth defect and had elevated MICs to azithromycin (8-fold), josamycin (8-fold) and erythromycin (16- to 32-fold). WGS did not identify other mutations likely to contribute to reduced macrolide susceptibility.

**Conclusions:**

A novel 23S rRNA gene mutation was identified in *M. genitalium*. This variation is found in *Mycoplasma hominis*, which is intrinsically resistant to certain macrolides. While this mutation has not been observed clinically in *M. genitalium*, these findings have expanded our understanding of resistance mechanisms within the Mollicutes, in particular the propensity for *M. genitalium* to develop resistance, even in low concentrations of antibiotic, and the interaction of azithromycin with the ribosome.

## Introduction

*Mycoplasma genitalium* is a sexually transmitted bacterium belonging to the Mollicutes class. It causes non-gonococcal urethritis in men and cervicitis and pelvic inflammatory disease in women.^1–4^ These bacteria lack a cell wall, making them resistant to multiple classes of antibiotics. While azithromycin as a single dose, recommended as the first-line treatment in some guidelines, was highly effective, the bacterium’s high propensity to develop resistance has resulted in a decrease in efficacy from 85% before 2009 to 67% in 2009–15.^5^ As a result, most guidelines now recommend an extended dose of azithromycin, after pre-treatment with doxycycline.^6–9^

**Figure 1.**
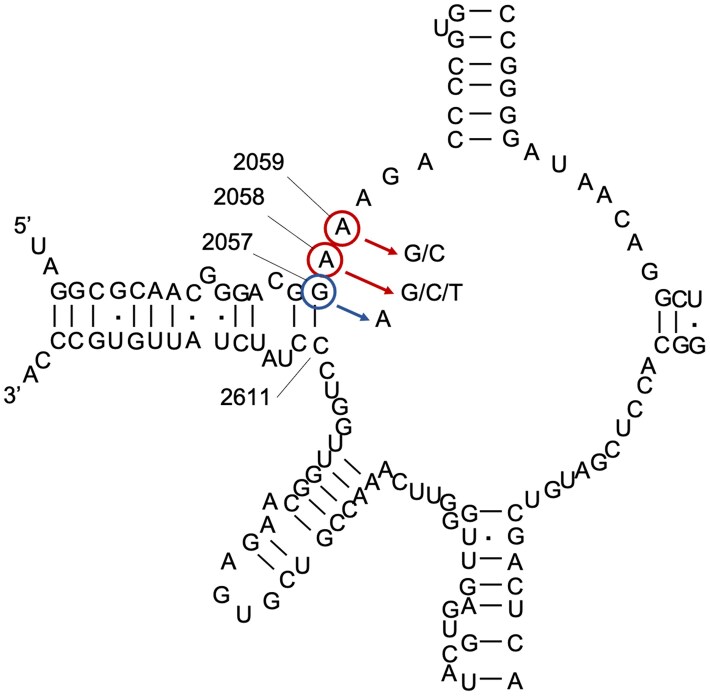
Domain V of the 23S rRNA of *M. genitalium*. Macrolide resistance mutations commonly found in clinical isolates are circled in red. The mutation induced in this study by exposure to azithromycin *in vitro* is circled in blue, with the corresponding base pair located at position 2611. The numbering used in this figure is based on *E. coli* sequence numbering. This figure has been drawn based on the structure presented in Pereyre *et al*.^10^

In *M. genitalium*, resistance to azithromycin is conferred by SNPs of the 23S rRNA gene at adenine 2058 (*Escherichia coli* numbering; mutation to cytosine, guanine or thymine) or adenine 2059 (mutation to guanine or cytosine) (Figure 1).^11^ Culture of clinical specimens is not routinely performed due to the difficulty in isolating *M. genitalium*. As a result, there has been limited MIC testing and analysis of azithromycin resistance *in vitro*. Of the few isolates carrying a A2058/A2059 mutation that have been tested, the MICs of azithromycin are ≥8 mg/L, which is over 1000-fold higher than the WT at 0.008 mg/L.^11–13^ In this study, *M. genitalium* was serially passaged in subinhibitory concentrations of azithromycin with the aim of generating mutants for further phenotypic characterization and to better understand how macrolide resistance develops in this bacterium. During this process, a novel 23S rRNA gene mutation was identified that conferred a 4–8-fold increase in MIC and is the subject of this study.

## Materials and methods

### Bacterial strains and culture methods

The laboratory-adapted *M. genitalium* strain G37 was used for all experiments (ATCC 33530). Bacteria were grown in Hayflick medium with the following composition: Bacto Heart Infusion Broth (2.5%), yeast extract (2.5%), phenol red (0.0003%), horse serum (20%), benzylpenicillin (0.003%), DNA sperm nuclei (0.0024%), and pH adjusted to 7.5 ± 0.2 (Media Preparation Unit, Peter Doherty Institute, Melbourne, Australia).

### Antibiotics and MIC assay

Antibiotics were purchased from Merck (NJ, USA), including azithromycin dihydrate (resuspended at 100 mg/mL in ethanol), josamycin (resuspended at 25 mg/mL in ethanol), and erythromycin (resuspended at 50 mg/mL in ethanol). Two-fold dilutions of azithromycin from 0.064 to 0.001 mg/L were used for the MIC assays and mutation induction.

Cultures were standardized to 10^3^ copies/µL in Hayflick medium for each individual MIC assay and for each iteration of mutation selection. MIC assays to select for mutants were performed initially in a 96-well Nunclon^™^ Delta Surface plate (Thermo Fisher Scientific, MA, USA), as described previously,^14^ before transitioning to 1.5 mL screw-cap microtubes (Sarstedt, Nümbrecht, Germany) as these were more convenient. After 10 days of incubation (estimated to be in the mid-late log phase of growth), bacterial concentration increased ∼100-fold to approximately 10^5^ copies/µL.

MIC assays for each macrolide were performed in 96-well Nunclon^™^ Delta Surface plates (Thermo Fisher Scientific). The concentration was measured using droplet digital PCR (ddPCR) targeting *mgpB*, as described below, and the MIC determined at a threshold of 90% growth inhibition. The well corresponding to the first antibiotic dilution below the MIC was used to inoculate the subsequent MIC assay. This was repeated until the development of resistance, indicated by an increase in MIC.

### ddPCR analysis of cultures

ddPCR was used to enumerate cultures and to detect the presence of 23S rRNA polymorphisms. Primers and probes can be found in Table 1. Enumeration of cultures was performed as described previously.^16^ To detect known 23S rRNA gene mutations, a new assay was developed using probes specific to each mutation. These were evaluated for specificity and cross-reaction using 500 bp synthetic DNA standards containing each of the known mutations [gBlocks, IDT Australia; Figure S1 (available as Supplementary data at *JAC* Online)], and further validated on samples containing known genetic variants (data not shown). An additional probe was designed to identify the novel mutation G2057A. For assay development, 0.25 µM of the respective 23S rRNA mutation probe was used while for analysis of cultures, 0.125 µM was used. The assays to detect each mutation were performed in independent reactions.

**Table 1.** PCR primers and probes used in this study

Name	Target	Purpose	Sequence (5′ → 3′)^a^	Reference
MgPa-355F	*mgpB*	Bacterial enumeration	GAGAAATACCTTGATGGTCAGCAA	Jensen *et al.*^15^
MgPa-432R	*mgpB*	Bacterial enumeration	GTTAATATCATATAAAGCTCTACCGTTGTTATC	Jensen *et al.*^15^
MgPa-380	*mgpB*	Bacterial enumeration	FAM-ACTTTGCAATCAGAAGGT-MGB	Jensen *et al.*^15^
Mg 23S-1986f	23S rRNA	Sanger sequencing, genotyping by ddPCR	GGTGTAACCATCTCTTGACTGTCTCGG^b^	Jensen *et al.*^11^
Mg 23S-2682r	23S rRNA	Sanger sequencing, genotyping by ddPCR	CGGTCCTCTCGTACTAGAAGCAAAG	Jensen *et al.*^11^
23S WT	23S rRNA	Genotyping by ddPCR	HEX-CAACGGGACG**GAA**AGACCCC-BHQ1	This study
23S A2058G	23S rRNA	Genotyping by ddPCR	FAM-CAACGGGACG**GGA**AGACCCC-BHQ1	This study
23S A2058C	23S rRNA	Genotyping by ddPCR	FAM-CAACGGGACG**GCA**AGACCCC-BHQ1	This study
23S A2058T	23S rRNA	Genotyping by ddPCR	FAM-CAACGGGACG**GTA**AGACCCC-BHQ1	This study
23S A2059G	23S rRNA	Genotyping by ddPCR	FAM-CAACGGGACG**GAG**AGACCCC-BHQ1	This study
23S A2059C	23S rRNA	Genotyping by ddPCR	FAM-CAACGGGACG**GAC**AGACCCC-BHQ1	This study
23S G2057A	23S rRNA	Genotyping by ddPCR	FAM-CAACGGGACG**AAA**AGACCCC-BHQ1	This study

^a^Bold bases indicate potential location of 23S rRNA mutations. Underlined bases indicate probe mismatch with WT (G37) sequence.

^b^Modified primer with an additional G at the 5′ end.

### Isolation of the G2057A mutant

To isolate mutants from a mixed population, a limiting dilution assay was performed as follows: the concentration of *M. genitalium* culture was determined using ddPCR then the culture was passed through a 25-gauge needle to dissociate clumps of bacteria.^17^ The culture was then diluted serially to reach a concentration of 0.5 copies/µL. Using a 96-well Nunclon^™^ Delta Surface plate (Thermo Fisher Scientific, Massachusetts, USA), 48 µL of Hayflick medium was added to each well, and 2 µL of the diluted culture. After growth was detected, ddPCR was used to confirm the genotype of the culture.

### Sequencing and data analysis

Dual-direction Sanger sequencing of the 23S rRNA genes was performed using the primers shown in Table 1 (Australian Genome Research Facility, Melbourne, Australia). Sequences were analysed using MEGA (version 10.1.7)^18,19^ and visualized using 4Peaks version 1.8 (Nucleobytes B.V., Amsterdam, the Netherlands).

To obtain whole-genome sequences, cultures of the mutant and the WT parent strain were centrifuged at 20 000 rcf for 30 min and the pellet was resuspended in 1× DNA/RNA Shield (Zymo Research, CA, USA) before extraction using the *Quick*-DNA^™^ HMW MagBead kit (Zymo Research). The sequence library was prepared using a ligation sequencing kit (SQK-NBD114.96; Oxford Nanopore Technologies, Oxford, UK) and sequenced using a FLO-MIN114 R10.4.1 flow cell on the MinION (Oxford Nanopore Technologies). Basecalling and adaptor trimming of reads was performed using Dorado version 0.7.2 (https://github.com/nanoporetech/dorado) with the super accuracy protocol version 5.0.0. Reads with a quality score of at least nine were filtered using nanoq version 0.10.0 (https://github.com/esteinig/nanoq) and passed reads were assembled *de novo* using Flye version 2.9.2 (https://github.com/fenderglass/Flye). The assembled contigs were polished with Medaka version 1.9.1 (https://github.com/nanoporetech/medaka) and the mutant whole genome was compared to the WT parent strain with the progressive Mauve algorithm^20^ plug-in in Geneious Prime version 2025.0.2 (Biomatters Ltd, Auckland, New Zealand).

## Results

### Induction of azithromycin resistance *in vitro*

**Figure 2.**
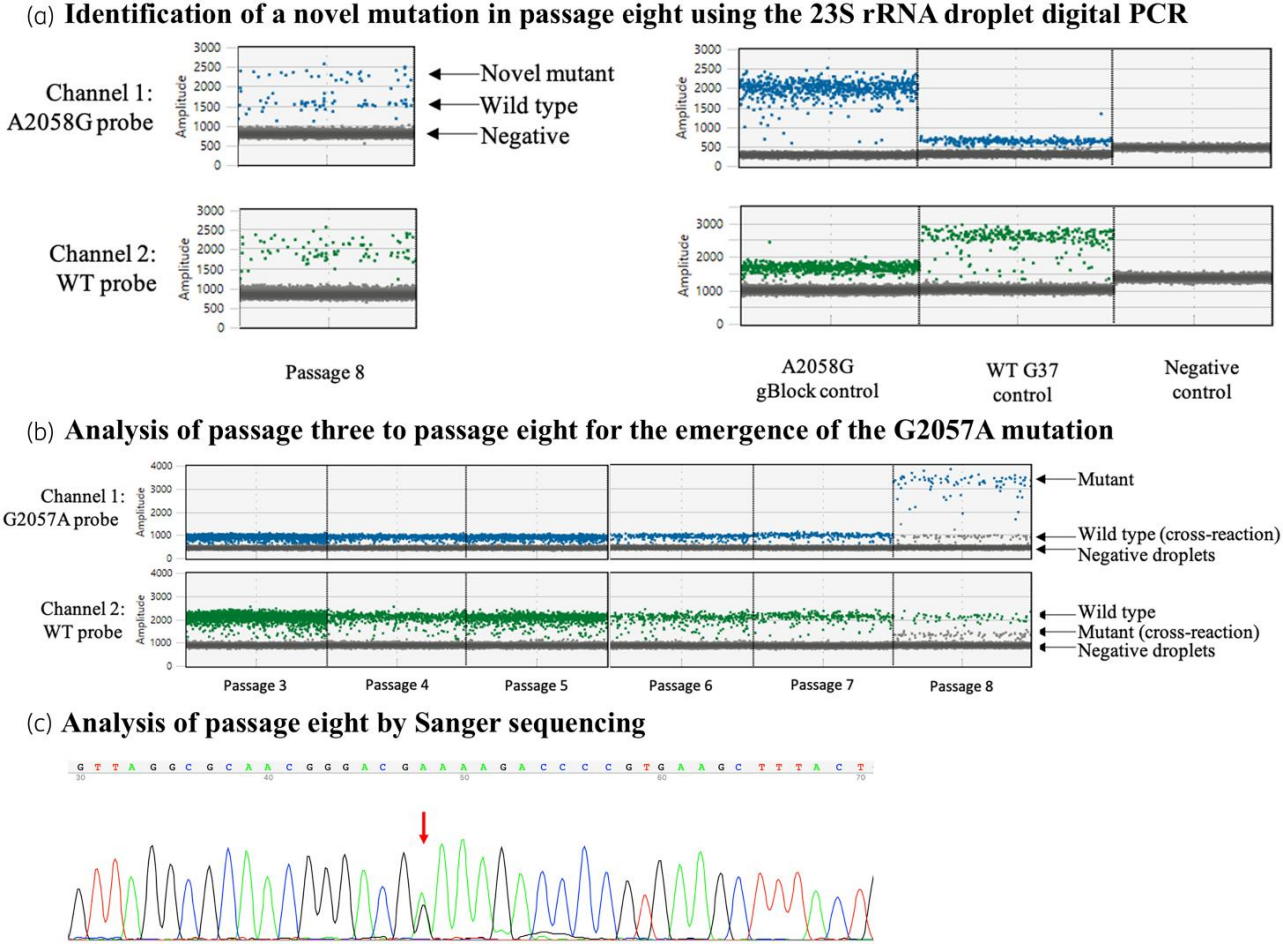
Detection of a novel mutation by ddPCR and Sanger sequencing. (a) Analysis of the mixed population found in passage eight. Controls are grouped on the right-hand side. Analysis with the A2058G probe (blue, top row) and WT probe (green, bottom row) is depicted. Probes targeting the other common mutations (Table 1) were also used with similar results (not shown). (b) Analysis of five passages prior to passage eight to determine when the mutation appeared. PCR was performed with the G2057A probe (blue, top row) and WT probe (green, bottom row). Respective DNA populations are indicated. (c) Sanger sequencing chromatogram of the 23S rRNA sequence of the mixed mutant and WT *M. genitalium* population. The red arrow corresponds to a dual peak of A/G at position 2057.

To select for macrolide resistance mutations *in vitro*, serial MIC assays were performed using azithromycin and the *M. genitalium* strain G37. Cultures were periodically screened by ddPCR to detect the presence of known mutations in the 23S rRNA gene. After the eighth passage there were no substantial increases in MIC values, which fluctuated around a median value of 0.008 mg/L. Analysis by ddPCR did not detect any known mutations at positions 2058 and 2059 that confer azithromycin resistance. However, from the culture at the eighth passage, an intermediate signal was detected with ddPCR using probes targeting each of the known 23S rRNA gene resistance variants (Table 1), indicative of a novel mutation (Figure 2a). This mutation was not present in previous passages (Figure 2b). Sequencing of the 23S rRNA gene in this culture identified a mixed population of bacteria with a WT 23S rRNA gene and a previously undescribed mutation (guanine-to-adenine transition at rRNA gene position 2057, G2057A; Figure 2c). Additional passages were performed to see whether the mutant would outgrow the WT population; however, after six passages, the proportion of mutant to WT did not change.

### Isolation of a G2057A mutant and MIC testing

A limiting dilution method was used to isolate the mutant from the WT G37. The isolates were confirmed to have a mutant 23S rRNA gene by ddPCR and Sanger sequencing. Using one clone, the azithromycin MIC was determined to be 0.032 mg/L. This was 8-fold higher than WT (0.004 mg/L; Table 2). The mutant clone was grown in antibiotic-free Hayflick medium to determine the stability of the mutation. The mutation remained after >10 passages, with no reappearance of WT (determined by ddPCR).

**Table 2.** Various macrolide MICs of *M. genitalium* G37 with a WT 23S rRNA and the mutant with a G2057A in the 23S rRNA

	MIC (mg/L)		
	AZM	JOS	ERY
WT 23S rRNA	0.004	0.02	0.04
G2057A 23S rRNA	0.032	0.16	0.64–1.28

AZM, azithromycin; JOS, josamycin; ERY, erythromycin.

MIC assays for the mutant were performed for josamycin and erythromycin to see if the mutation conferred resistance to additional macrolides. The MICs for the mutants were 8-fold and 16–32-fold higher than for WT, respectively (Table 2).

### Comparison of the growth rate between the azithromycin-resistant mutant and WT

**Figure 3.**
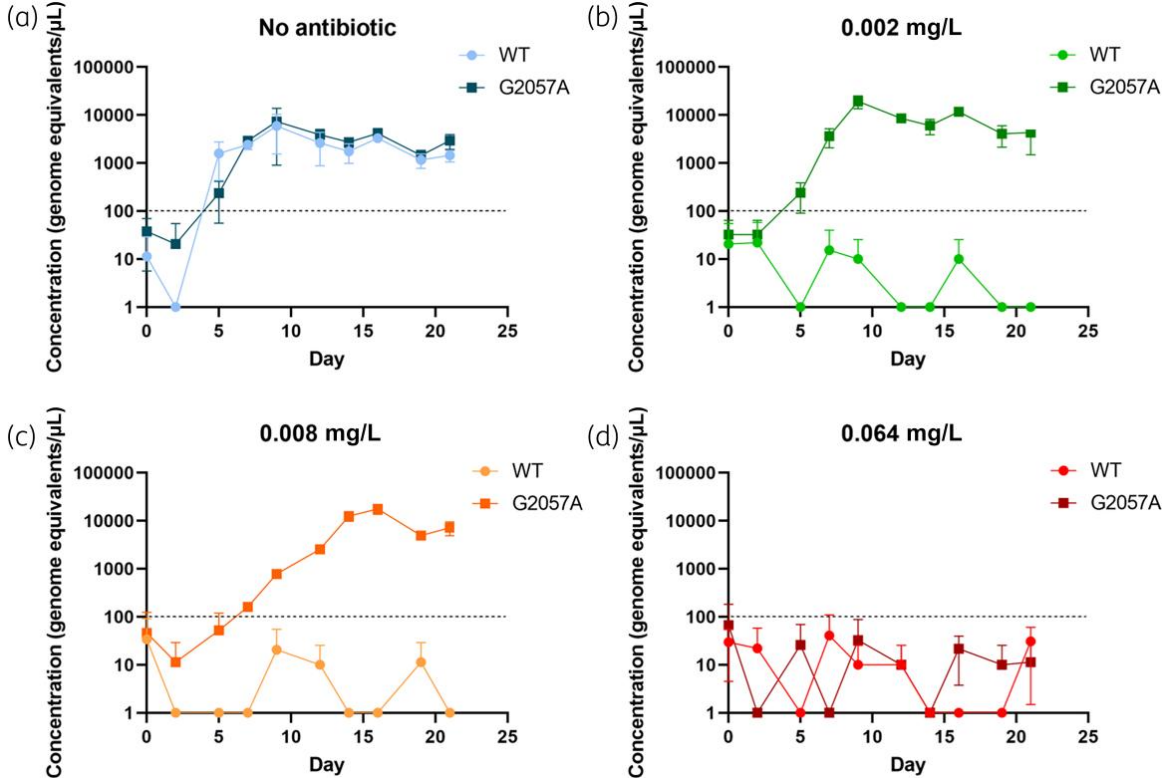
Growth of the WT G37 and G2057A mutant in Hayflick medium with no azithromycin (a), 0.002 mg/L azithromycin (b), 0.008 mg/L azithromycin (c) and 0.064 mg/L azithromycin (d). Cultures were grown in triplicate and enumerated using the 23S rRNA ddPCR assay. Error bars represent 1SD. The dotted line indicates the target starting concentration for cultures.

The growth of the G2057A mutant was examined to see if the mutation had an impact on bacterial fitness. The WT and G2057A mutant were grown together in cultures containing no azithromycin, and with azithromycin (0.002, 0.008, and 0.064 mg/L) and enumerated by ddPCR. The WT was only able to grow in the culture with no azithromycin, whereas the G2057A mutant grew in azithromycin up to a concentration of 0.008 mg/L, albeit the exponential phase was longer in higher concentrations of azithromycin (Figure 3). Both the WT and G2057A mutant reached the same maximum growth density, regardless of antibiotic concentration for the mutant.

### Sequence analysis of the 23S rRNA gene of the G2057A mutant

Sanger sequencing of the whole 23S rRNA gene was performed to identify if there were other mutations in the gene, such as a compensatory mutation in the cytosine residue (C2611) that pairs with the mutated G2057 (Figure 1). No mutations were identified.

To determine if the 23S rRNA gene change was the sole mutation contributing to phenotypic resistance we sequenced the whole genome of the mutant and compared it with that of the parent strain. There were some single nucleotide polymorphisms and indels (Table S1); however, these are unlikely to contribute to macrolide resistance.

## Discussion

Antibiotic resistance is an ongoing concern, especially in *M. genitalium*, where resistance to azithromycin, a commonly used antibiotic, is as high as 80% in some communities such as MSM attending sexual health clinics.^21^ While mutations affecting A2058 and A2059 of the 23S rRNA gene have been documented, the impact of individual mutations on azithromycin resistance is not fully understood. In this study, serial passaging of the laboratory strain of *M. genitalium* in subinhibitory concentrations of azithromycin selected for a novel 23S rRNA mutation, G2057A. This mutation conferred an 8-fold increase in azithromycin MIC and 8–32-fold increase in MICs of other macrolides. The G2057A mutant was able to grow in cultures with up to 0.008 mg/L azithromycin, albeit at a slower rate than in no azithromycin.

This study enhances our understanding of macrolide resistance mechanisms in the Mollicutes. While the G2057A 23S rRNA gene mutation has not previously been described in *M. genitalium*, this sequence naturally occurs in *Mycoplasma hominis*, *Mycoplasma fermentans* and *Mycoplasma pulmonis*, three species with higher intrinsic azithromycin resistance (≥2 mg/L).^22^ In contrast, *M. genitalium* and *Mycoplasma pneumoniae* both naturally have a guanine at position 2057 and are susceptible to 14- and 15-membered macrolides, such as erythromycin and azithromycin.^22,23^ Most notably, *M. hominis* is intrinsically resistant to 14- and 15-membered macrolides, but susceptible to 16-membered macrolides, such as josamycin, and lincosamides. Consistent with the findings in *M. hominis*, the *M. genitalium* G2057A mutant had increased MICs of 14-membered ring (erythromycin) and 15-membered ring (azithromycin) macrolides.

In contrast to A2058/A2059 mutations, which confer a large increase in MIC (approximately 1000-fold higher than WT), the G2057A mutation conferred a lower level of resistance to azithromycin (8-fold higher). The limited increase in MIC attributed to the G2057A mutation is similar to what is observed with fluoroquinolone-resistance mutations affecting ParC; for example, the ParC S83I change leads to a modest 32-fold (geometric mean) increase in MIC to ≥1 mg/L.^12,24^ This may explain why A2058/A2059 mutations were not selected in this study, as the concentration of azithromycin was too low; it is also possible that A2058/A2059 mutations have a higher fitness cost or are less likely to develop compared with G2057 mutations *in vitro*.

Conversely, it is likely that G2057A mutations are not observed clinically as the current dosage levels used for clinical treatment are sufficient for clearance of *M. genitalium* harbouring this mutation. This was corroborated by growth in 0.002 and 0.008 mg/L azithromycin, but not 0.064 mg/L; at 0.008 mg/L of azithromycin, the G2057A mutant was slower-growing, taking longer to reach the maximum growth density, indicating some inhibition. Therefore, it is probable that G2057A mutations are less likely to be maintained in circulating populations of *M. genitalium* as they will be eliminated by treatment, while A2058/A2059 mutations will be enriched, leading to wider dissemination of these mutations. It is also likely that the lack of G2057A mutations identified among clinical samples is because this mutation is unlikely to develop or happen randomly *in vivo*.

Macrolides inhibit protein synthesis by interfering with the polypeptide exit tunnel of the large subunit of the bacterial ribosome, preventing the passage of nascent polypeptides.^25,26^ The key binding sites for macrolides are A2058 and A2059 in domain V of the peptidyl transferase loop.^26–28^ The base-pairing between G2057 and C2611 is important for binding of some macrolides by keeping a closed conformation in the peptidyl transferase loop.^29^ In this study, disrupted base pairing with G2057A/C2611 had varied impact on macrolide binding; for erythromycin, there was a 16–32-fold increase in MIC, whereas for azithromycin and josamycin there was an 8-fold increase. This observation is similar to a study that selected for macrolide-resistant *M. pneumoniae in vitro*, where a mutant was isolated with a C2611A transition, leading to modest increases in MIC of erythromycin (33-fold), but small changes for azithromycin (4-fold) and josamycin (<2-fold).^30^ In other bacteria, resistance to macrolides can also be conferred by mutations in the L4 and L22 ribosomal proteins, which change the conformation of the ribosome.^31,32^ Mutations in these two proteins were selected for *in vitro* in *M. pneumoniae*, but the contribution to clinical resistance is unclear.^30,33–36^ The role of L4 and L22 in macrolide resistance has received limited investigation in *M. genitalium* with no associations identified,^11,37^ and variations were not observed in the G2057A mutant.

This study has limitations. First, the laboratory strain of G37 was not single-colony cloned before performing mutant induction, therefore, the starting bacterial population potentially contained some degree of genetic variation (as evident in the whole genome sequence). While this may complicate the attribution of phenotype to a single mutation, it more accurately replicates the diversity of an inoculum in a normal infection. Second, this study only used azithromycin to select for resistance-conferring mutations, which is commonly used for treatment of *M. genitalium* and other sexually transmitted infections. It is known that not all macrolides will select for the same resistance mutations;^30^ whether other macrolide antibiotics may inadvertently select for resistance in *M. genitalium* is unknown, and future studies could use other macrolide antibiotics to induce resistance. Finally, WGS identified other variations within the genome compared with the parent strain used to start the experiment; however, these are unlikely to contribute to macrolide resistance. It is likely that an accumulation of random variations occurred in this strain prior to the development of a beneficial azithromycin resistance mutation.

In summary, this study identified a novel G2057A mutation in *M. genitalium* conferring modest azithromycin resistance. While the *in vitro*-derived G2057A mutation has not been identified in clinical specimens, it has provided a new perspective into resistance mechanisms more broadly in the Mollicutes.

## Supplementary Material

dkaf174_Supplementary_Data

## Supplementary data

Figure S1 and Table S1 are available as Supplementary data at *JAC* Online.
