# Supplementary material for: A novel azithromycin resistance mutation in Mycoplasma genitalium induced in vitro
Source: J Antimicrob Chemother. 2025 Jun 4;80(7):2044–50. doi: 10.1093/jac/dkaf174 (PMC12209806; doi:10.1093/jac/dkaf174)
Supplement: dkaf174_Supplementary_Data [file dkaf174_supplementary_data.zip › Table S1.docx]

| **Gene/Protein** | **Function** | **Location on G37*** | **Nucleotide change** | **Amino acid change** |
| --- | --- | --- | --- | --- |
| DEAD/DEAH box helicase | RNA-binding proteins involved in RNA metabolism | 21,640 | A deletion | N/A (repeated A region) |
| Non-coding region | N/A | 36,794 | A deletion | N/A |
| mgpC | Cytoadherence protein | 169,477–9 | AGT deletion | S deletion |
| 23S rRNA | Part of ribosome | 173,797 | G to A | N/A |
| P110 | Adhesin protein | 227,129–31 | AGT deletion | S deletion |
| FAD-dependent oxidoreductase | Catalysis of redox reactions | 334,429 | T to C | Silent |
| Hypothetical protein | Unknown | 430,014–6 | CTA deletion | L deletion |

Table S1. Summary of nucleotide changes in the G2057A mutant (whole genome analysis)

*GenBank accession NC_000908
