# Supplementary figures and images for: A novel azithromycin resistance mutation in Mycoplasma genitalium induced in vitro
Source: J Antimicrob Chemother. 2025 Jun 4;80(7):2044–50. doi: 10.1093/jac/dkaf174 (PMC12209806; doi:10.1093/jac/dkaf174)

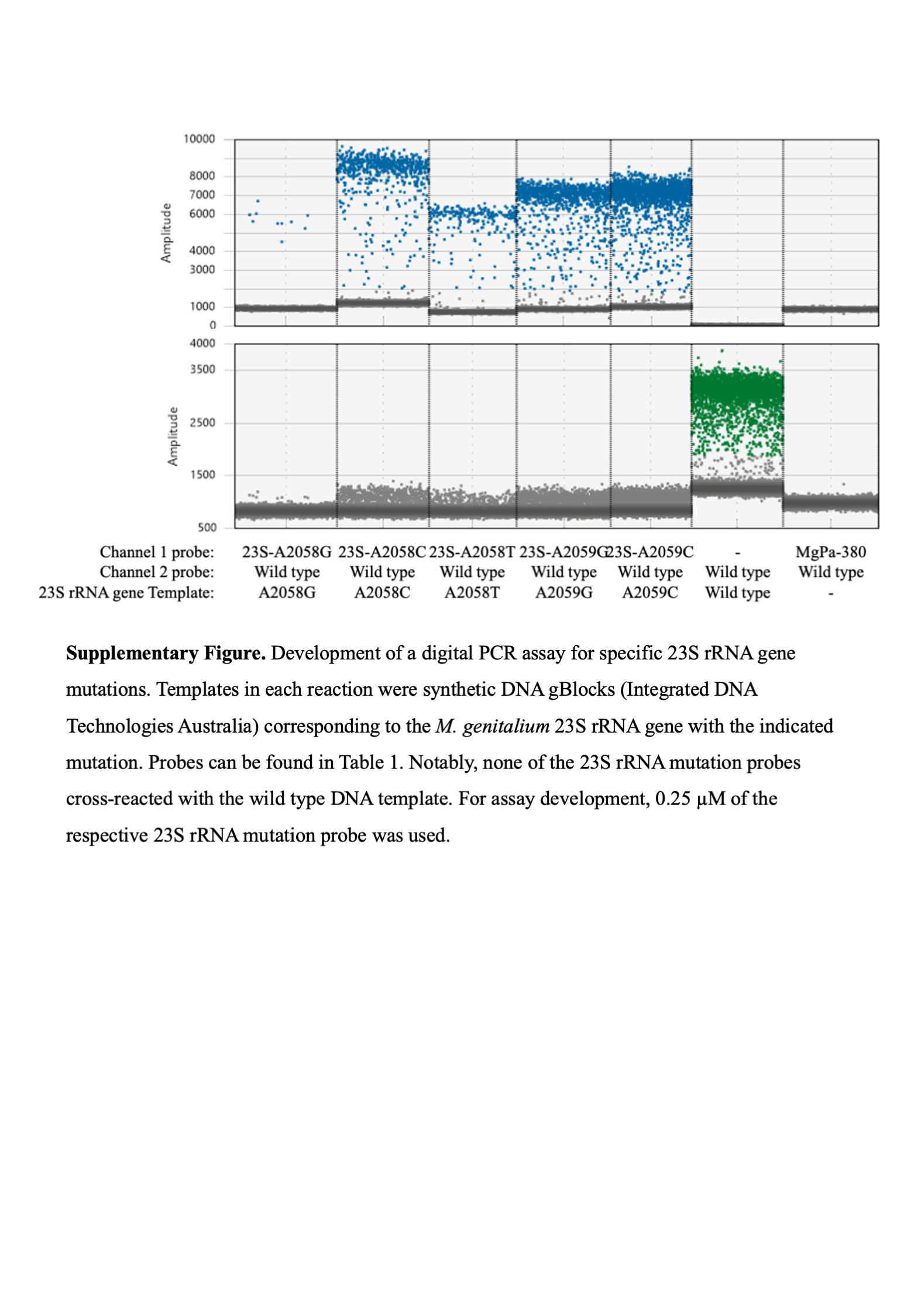

Supplement: dkaf174_Supplementary_Data [file dkaf174_supplementary_data.zip › Figure S1 legend.tiff]
